# A Pay-It-Forward Approach to Improve Chlamydia and Gonorrhea Testing Uptake Among Female Sex Workers in China: Venue-Based Superiority Cluster Randomized Controlled Trial

**DOI:** 10.2196/43772

**Published:** 2023-03-02

**Authors:** Weiming Tang, Yewei Xie, Mingzhou Xiong, Dan Wu, Jason J Ong, Teodora Elvira Wi, Bin Yang, Joseph D Tucker, Cheng Wang

**Affiliations:** 1 Dermatology Hospital of Southern Medical University Guangzhou China; 2 Southern Medical University Institute for Global Health Guangzhou China; 3 University of North Carolina at Chapel Hill Project-China Guangzhou China; 4 Health Service & System Research programme Duke-NUS Medical School Singapore Singapore; 5 Department of Clinical Research Faculty of Infectious and Tropical Diseases London School of Hygiene and Tropical Medicine London United Kingdom; 6 West China School of Public Health Sichuan University Chengdu China; 7 Central Clinical School Faculty of Medicine Monash University Melbourne Australia; 8 Department of Global HIV, Hepatitis and STI Programmes World Health Organization Headquarters Geneva Switzerland

**Keywords:** pay-it-forward, chlamydia, gonorrhea, testing, female sex workers, women, China, cost, stigma, prevention, community, HIV, care, STD, implementation, research

## Abstract

**Background:**

Regular chlamydia and gonorrhea testing are essential for key populations, such as female sex workers (FSWs). However, testing cost, stigma, and lack of access prevent FSWs in low- and middle-income countries from receiving chlamydia and gonorrhea testing. A social innovation to address these problems is “pay it forward,” where an individual receives a gift (free testing) and then asks whether they would like to give a gift to another person in the community.

**Objective:**

This cluster randomized controlled trial examined the effectiveness and cost of the pay-it-forward strategy in increasing access to chlamydia and gonorrhea testing among FSWs in China.

**Methods:**

This trial integrated a pay-it-forward approach into a community-based HIV outreach service. FSWs (aged 18 years or older) were invited by an outreach team from 4 Chinese cities (clusters) to receive free HIV testing. The 4 clusters were randomized into 2 study arms in a 1:1 ratio: a pay-it-forward arm (offered chlamydia and gonorrhea testing as a gift) and a standard-of-care arm (out-of-pocket cost for testing: US $11). The primary outcome was chlamydia and gonorrhea test uptake, as ascertained by administrative records. We conducted an economic evaluation using a microcosting approach from a health provider perspective, reporting our results in US dollars (at 2021 exchange rates).

**Results:**

Overall, 480 FSWs were recruited from 4 cities (120 per city). Most FSWs were aged ≥30 years (313/480, 65.2%), were married (283/480, 59%), had an annual income <US $9000 (301/480, 62.7%), and had never been tested for chlamydia (401/480, 83.5%) or gonorrhea (397/480, 82.7%). Chlamydia and gonorrhea test uptake in the pay-it-forward and standard-of-care arms were 82% (197/240) and 4% (10/240), respectively, with an adjusted proportion difference of 76.7% (lower bound 95% CI 70.8%). All those who tested positive were referred to and received treatment at local sexually transmitted infection clinics. This finding was consistent when adjusting for marital status, income, inconsistent condom use during commercial sex in the last 3 months, and HIV testing history. Among 197 women who received tests in the pay-it-forward arm, 99 (50.3%) donated money, with a median donation of US $1.54 (IQR 0.77-1.54). The economic cost per person tested was US $568.71 for standard of care and US $43.20 for pay it forward.

**Conclusions:**

The pay-it-forward strategy has the potential to enhance chlamydia and gonorrhea testing for Chinese FSWs and may be useful for scaling up preventive services. Further implementation research is needed to inform the transition of pay-it-forward research into practice.

**Trial Registration:**

Chinese Clinical Trial Registry ChiCTR2000037653; https://www.chictr.org.cn/showprojen.aspx?proj=57233

## Introduction

Chlamydia and gonorrhea are two of the most common bacterial sexually transmitted infections (STIs). Each year, there are an estimated 129 million new cases of chlamydia and 82 million new cases of gonorrhea globally, and the majority are asymptomatic [1]. Female sex workers (FSWs) face a high burden of chlamydia and gonorrhea, with an estimated prevalence of 4% to 15% and 1% to 11%, respectively, while FSWs in low- and middle-income countries (LMICs) have a higher prevalence of chlamydia and gonorrhea [2-6].

Testing, especially for high-risk women, is essential for chlamydia and gonorrhea control and the reduction of adverse sexual and reproductive complications [1,7]. The World Health Organization guidelines recommend regular chlamydia and gonorrhea molecular assay testing for sex workers in LMICs [8]. However, despite a high disease burden, few FSWs receive chlamydia and gonorrhea testing in LMICs. For example, less than one-tenth of Chinese FSWs have ever received chlamydia and gonorrhea testing [9]. First, although chlamydia and gonorrhea testing are available in many Chinese hospitals, the out-of-pocket cost for testing is relatively high (US $11) and not covered by most public or private health insurance programs [10]. Given that more than half of Chinese FSWs have an average monthly income of less than US $1000 [11], financial limitations may remain a significant barrier preventing FSWs from accessing chlamydia and gonorrhea testing. Second, current STI prevention services in many LMICs, including China, mainly focus on HIV and syphilis [1]. These well-established HIV and syphilis testing systems do not typically integrate other STI testing [12]. China does not have a screening strategy for chlamydia or gonorrhea for high-risk and other vulnerable populations, nor does it have widespread programs supporting chlamydia and gonorrhea testing. Third, peer factors relating to mistrust in services may decrease interest in routine service delivery [13]. This mistrust in STD services might dramatically reduce access to STD care services, including chlamydia and gonorrhea testing in clinical settings [14]. A lack of community engagement might be an essential reason for mistrust [15].

A pay-it-forward health approach may help promote test uptake among FSWs. In a pay-it-forward approach, one person receives a gift (eg, a free STI test, alongside community-engaged messages) and then is asked whether they would like to donate money to another person [16]. This social innovation has the potential of not only reducing financial barriers but also increasing trust and community engagement in health services by increasing community ownership. Two studies among Chinese men who have sex with men (MSM) found that the pay-it-forward approach could substantially increase chlamydia and gonorrhea testing uptake [10,17]. However, these studies only focused on MSM in 2 cities, limiting the generalizability of the findings.

This multisite cluster randomized controlled trial (RCT) aimed to evaluate the effectiveness and cost of a pay-it-forward strategy in increasing dual chlamydia and gonorrhea testing among FSWs in 4 cities of Guangdong Province, China, compared with a standard fee-based system.

## Methods

### Study Design

This study was a superiority cluster RCT to test whether the pay-it-forward intervention was superior to the standard of care in increasing chlamydia and gonorrhea testing. The study was conducted in 4 cities (Maoming, Yunfu, Yangjiang, and Yingde), with 1 cluster per city, in Guangdong Province, China. These cities were chosen based on local capacity and the availability of ongoing public health community-based outreach programs for FSWs. The reason for choosing a cluster RCT design was that many FSWs in the same city would be recruited from the same venues, and an individual RCT design may have caused contamination between different arms.

The local Centre for Disease Control and Prevention outreach team included a nurse, an STI physician from a local STI clinic, and a public health practitioner (as a health educator). The outreach team routinely provided venue-based sexual health care services to local FSWs. These outreach teams have long-standing relations with FSWs and provide comprehensive health services, including condom promotion, reproductive health services, STI counseling, symptomatic STI treatment, and on-site HIV testing.

We estimated we needed 480 participants from the 4 clusters (120 from each city). Participants were recruited after randomization of the selected cluster. Our previous studies among MSM indicated that the pay-it-forward approach is superior to the standard of care by a 20% margin. We kept the same margin for this superiority trial. The sample size was determined using the following assumptions: the testing rate of the null hypothesis for the pay-it-forward group was set at 30% (the null hypothesis was that the pay-it-forward group would increase the testing rate by at least 10%), the testing rate of the alternative hypothesis was set at 50%, and the testing rate of the null hypothesis for the standard of care group was set at 20%, with an interclass correlation of 0.1.

### Ethical Considerations

Study approval was gained from the ethics review committees of the Southern Medical University Dermatology Hospital (#2020018). The CONSORT (Consolidated Standards Of Reporting Trials) cluster-extension guidelines were used to report the findings from this study (Multimedia Appendix 1). This study was registered at the Chinese Clinical Trial Registry (ChiCTR2000037653). All the participants who participated in the survey signed an informed consent form. The informed consent form included a statement that study data were anonymous or would be deidentified.

### Participants

Many sex workers in China do not routinely seek clinic-based services, so we integrated this study within an ongoing HIV- and syphilis-testing outreach program. The study participants were recruited by having the outreach team, including a study staff member, go to their workplaces during working hours (usually in the evening). Before this study, the local outreach team mapped sex-work venues in each study site according to geographic area and type of venue. Convenience sampling was used to enroll FSWs at venues in each city. We categorized the sex work venues into high and low tiers based on the clients’ socioeconomic status and the cost for clients of commercial sex at these venues [18]. Low-tier venues included foot-bath shops, hair salons, massage parlors, roadside restaurants, roadside shops, guesthouses, streets, and public outdoor places. High-tier venues included karaoke bars, hotels, saunas, and nightclubs.

The inclusion criteria were as follows: sex at birth was female, age was 18 years or older, the participant self-reported as having engaged in transactional sex to obtain money or property in the past month in the sampling city, the participant had not previously participated in a research project related to gonorrhea or chlamydia testing, and the participant had not been tested for gonorrhea or chlamydia in the past 12 months.

The study was anonymous, but the eligible participants had to provide a cell-phone number for testing results notification. The phone number was also used for deduplication. If the participants had positive test results for as either chlamydia or gonorrhea, they were provided free treatment at a local STI clinic, regardless of whether they had symptoms.

### Randomization and Masking

Before participant recruitment, the 4 clusters were randomized into the 2 study arms in a 1:1 ratio. Participants recruited from Yunfu and Yingde cities were randomized into the pay-it-forward intervention group, and participants recruited from Maoming and Yangjiang cities were randomized into the control group. The reason for adopting a cluster randomized allocation method was the strong correlation between FSW test uptake in local areas of a city [19]. In addition, many of the FSWs were recruited from the same study sites. Individual-level randomization would have induced strong contamination between the study groups. We did not randomize the participants based on venue, as many street-based FSWs work alone.

### Procedures

The intervention was adapted from our previous trials, and a pilot intervention was implemented among 40 FSWs in Guangzhou, China, before the study was carried out.

**Figure 1:**
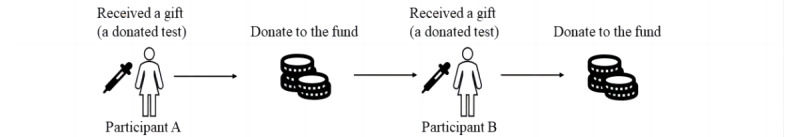
The pay-it-forward approach.

The pay-it-forward strategy was implemented in the intervention group. The study staff visited FSW workplaces in the selected cities in the pay-it-forward arm. First, after providing HIV and syphilis testing services, the study staff briefly introduced the reasons for chlamydia and gonorrhea testing, explained the pay-it-forward strategy, informed the participants of the market price of chlamydia and gonorrhea testing (approximately US $11 for both the intervention and control groups), and communicated that the testing cost had been covered by donations from other FSWs who care about their health. Therefore, each FSW could decide whether to receive donated chlamydia and gonorrhea testing services. If they chose to receive the test, they self-collected urine samples in a private room at their workplace. After that, they were asked whether they would voluntarily donate any amount to other FSWs for testing. A combination of study funds and donations from previous participants covered all testing costs for the intervention group. The donations were voluntary and were given after the testing. The key concepts of the pay-it-forward model are depicted in Figure 1.

The researchers also introduced chlamydia and gonorrhea testing to the standard-of-care arm without mentioning the pay-it-forward intervention. Participants who wished to be tested for chlamydia and gonorrhea in the standard-of-care arm self-collected their samples for testing in outreach settings. They were only required to pay a reduced price for their testing (approximately US $11). The participants in both arms were offered an incentive of US $7.50 for their participation and time spent in the research.

Participants in each group were invited to complete a short baseline questionnaire to collect information on their sexual history, testing history, attitudes toward the test, and the psychosocial environment in which they lived. We asked participants whether they used a condom during their last commercial sex act and whether they consistently used a condom during commercial sex in the previous months (consistent use meant using a condom every time).

All samples were transported to the laboratory at the Dermatology Hospital of Southern Medical University in Guangzhou for nucleic acid amplification testing (Cobas 4800 CT/NG Test Kits; Roche Molecular Systems).

### Outcomes

The primary outcome was the uptake of dual chlamydia and gonorrhea testing immediately after the intervention, measured during the study visit. The secondary outcome was the cost per person tested and the incremental cost-effectiveness ratio (ICER) between the 2 study arms. We calculated the financial and economic costs from a health care provider perspective, including direct medical expenses related to testing and personnel time. Costs are presented in US dollars (at 2021 exchange rates). We calculated the cost per person tested by dividing each arm’s total cost by the number of FSWs tested. The ICER was also calculated as the incremental cost of the intervention arm compared to the control arm divided by the incremental effectiveness (per person tested).

### Statistical Analysis

All statistical analyses were conducted at the cluster level. We first compared the sociodemographic characteristics of the participants in the 2 study arms. The generalized estimating equations (GEEs) with a binomial distribution and an identity link function were used to compare the proportion of chlamydia and gonorrhea tests in the different arms, reported as the crude probability difference. The equal correlation structure was specified as the correlation structure within groupings. Additionally, due to the small number of clusters in this study, the Mancl and DeRouen bias-corrected covariance estimator was applied to our generalized estimating equations to minimize the risk of a type I error [20,21]. A sensitivity analysis was also conducted, exploring the effects when considering FSW workplaces as clusters in the GEE model corrected by the Kauermann-Carroll method (Multimedia Appendix 2, Table S1) [21,22]. In the adjusted model, we further adjusted for the following variables: marital status and past experience of testing for HIV, chlamydia, or gonorrhea. These covariates were considered potential confounders, and some were not balanced between the 2 study groups because of the small cluster size. As this study used a superiority trial design, only one side of the CI is reported [17].

A subgroup analysis was performed to investigate the probability difference categorized by age, consistent condom use during commercial sex in the past 3 months, history of ever having been tested for HIV, and whether the participant worked in a high-tier workplace. These analyses were conducted with Stata (version 16; Stata Corp). The economic evaluation was performed using TreeAge Pro 2021 (TreeAge Software Inc).

## Results

### Study Participants

**Figure 2:**
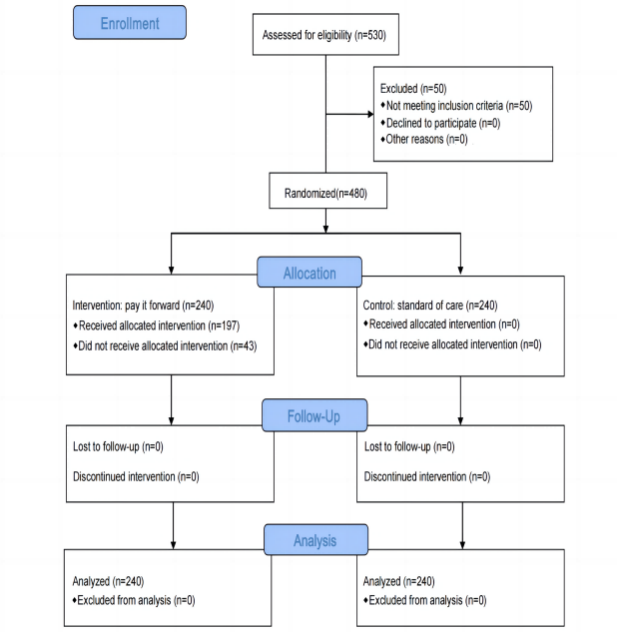
Flow diagram of participant inclusion.

This study was implemented from August 12, 2020, to November 15, 2020. We screened 530 female sex workers and recruited 480 who met the screening criteria. Fifty women were excluded from the study (2 were younger than 18 years, and 48 self-reported not engaging in commercial sex; Figure 2); 240 participants from 2 clusters were included in each study group. All enrolled women had data on the primary outcome.

Table 1 shows the participants’ sociodemographic characteristics, sexual behavior, and past STI testing history. Most women were aged 30 years or older (313/480, 65.2%), were married (283/480, 59%), and had a middle-school or lower education level (395/480, 82.3%). The majority worked at high-tier venues (311/480, 64.8%) and had an annual income of less than US $9000 (301/480, 63.6%). Nearly half of the women (235/480, 49%) had worked for longer than one year at their workplace. Around one-third of the participants (179/480, 37.3%) had used a condom consistently during commercial sex in the past 3 months. Many (290/480, 60.4%) women had never been tested for HIV, and the majority had never been tested for gonorrhea (397/480, 82.7%) or chlamydia (401/480, 83.5%).

**Table 1:** Participant characteristics by testing scenario among female sex workers in Guangdong, China, in 2020.

Characteristics		Total (N=480)	Pay it forward (n=240)	Standard of care (n=240)
**Age (years), n (%)**				
	<30	167 (34.8)	86 (35.8)	81 (33.8)
	>30	313 (65.2)	154 (64.2)	159 (66.3)
Age (years), mean (SD)		34.7 (9.0)	34.6 (9.7)	34.75 (8.2)
**Marital status, n (%)**				
	Never married	114 (23.8)	62 (25.8)	52 (21.7)
	Married	283 (59)	119 (49.6)	164 (68.3)
	Divorced or widowed	83 (17.3)	59 (24.6)	24 (10)
**Highest education, n (%)**				
	Middle school or below	395 (82.3)	191 (79.6)	204 (85)
	High or vocational school	76 (15.8)	45 (18.8)	31 (12.9)
	College or above	9 (1.9)	4 (1.7)	5 (2.1)
**Annual income (US $), n (%)**				
	<1800	2 (0.4)	1 (0.4)	1 (0.4)
	1800-6000	299 (62.3)	158 (65.8)	141 (58.7)
	>9000	179 (37.3)	81 (33.8)	98 (40.9)
**Type of workplace, n (%)**				
	High-tier venues^a^	311 (64.8)	156 (64.2)	157 (65.4)
	Low-tier venues^b^	169 (35.2)	86 (35.8)	83 (34.6)
**Time working at current workplace (months), n (%)**				
	<1	32 (6.7)	22 (9.2)	10 (4.2)
	1-6	121 (25.2)	75 (31.3)	46 (19.2)
	7-12	92 (19.2)	50 (20.8)	42 (17.5)
	>12	235 (49)	93 (38.8)	142 (59.2)
**Used condom during last commercial sex act, n (%)**				
	Yes	358 (74.6)	148 (61.7)	210 (87.5)
	No	122 (25.4)	92 (38.3)	30 (12.5)
**Frequency of condom use during commercial sex in the past 3 months, n (%)**				
	Nonuse	67 (14)	57 (23.8)	10 (4.2)
	Sometimes (less than half the time)	120 (25)	48 (20)	72 (30)
	Often (more than half the time)	114 (23.8)	29 (12.1)	85 (35.4)
	Consistent use	179 (37.3)	106 (44.2)	73 (30.4)
**HIV testing frequency in the past 2 years, n (%)**				
	Never tested	290 (60.4)	195 (81.3)	95 (39.6)
	<Once every 2 years	70 (14.6)	19 (7.9)	51 (21.3)
	Once a year or more frequently	120 (25)	26 (10.9)	94 (39.2)
**Gonorrhea testing frequency in the past 2 years, n (%)**				
	Never tested	397 (82.7)	219 (91.3)	178 (74.2)
	<Once every 2 years	53 (11)	8 (3.3)	45 (18.8)
	Once a year or more frequently	30 (6.2)	13 (5.4)	17 (7.1)
**Chlamydia testing frequency in the past 2 years, n (%)**				
	Never tested	401 (83.5)	226 (94.2)	175 (72.9)
	<Once every 2 years	52 (10.8)	4 (1.7)	48 (20)
	Once a year or more frequently	27 (5.6)	10 (4.2)	17 (7.1)
Size of donation (US $), median (IQR)		N/A^c^	10 (5-10)	N/A
**Gonorrhea test result^d^, n (%)**				
	Positive	3 (1.5)	3 (1.5)	0 (0)
	Negative	200 (98.5)	194 (98.5)	10 (100)
**Chlamydia test result^d^, n (%)**				
	Positive	34 (16.7)	34 (17.3)	0 (0)
	Negative	169 (83.3)	163 (82.7)	10 (100)

^a^High-tier venues included karaoke bars, hotels, saunas, and night clubs.

^b^Low-tier venues included foot-bath shops, hair salons or barbershops, massage parlors, roadside restaurants, guesthouses, streets, and public outdoor places.

^c^N/A: not applicable.

^d^For these values, n=203, n=197, and n=10 for the total, pay-it-forward, and standard-of-care groups, respectively.

### Testing Uptake

Overall, 197 of 240 (82.1%) women in the pay-it-forward arm and 10 of 240 (4.2%) women in the standard-of-care arm received dual chlamydia and gonorrhea testing (Table 2). In the pay-it-forward arm, 34 of 197 (17.3%) women were diagnosed with chlamydia, and 3 of 197 (1.5%) were diagnosed with gonorrhea. No chlamydia or gonorrhea was detected among women who received a standard-of-care test. All women who received a test were informed of the test results via text message (for women who tested negative) or phone call (for women who tested positive for a chlamydia or gonorrhea infection). Test-positive women were referred to local STI clinics for free treatment. The reasons for testing or not testing in the pay-it-forward arm and the program’s perceived benefits are summarized in Multimedia Appendix 3, Table S4.

The GEE model showed that the pay-it-forward intervention was associated with a 77.9% (lower-bound 95% CI 70.3%) increase in test uptake probability compared to the standard-of-care arm. After adjusting for marital status and past testing experience for HIV, chlamydia, and gonorrhea, the outputs were consistent with the crude results (adjusted probability difference 76.7%, lower-bound 95% CI 70.8%; Table 2).

Table 3 shows findings from prespecified subgroup analyses. The difference between the control and pay-it-forward arms was most significant among FSWs who had used condoms consistently during commercial sex in the previous 3 months (86%, 95% CI 78.6%-93.5%) and women older than 30 years (82%, 95% CI 75.7%-88.2%). The difference between the control and pay-it-forward arms was 4% (95% CI –8.6% to 16.6%) for a past history of HIV testing (no vs yes) and –5.1% (95% CI –16.3% to 6%) for workplace tier.

**Table 2:** The proportion of participating female sex workers tested for chlamydia and gonorrhea in Guangdong, China, in 2020 (N=480). The model was adjusted for marital status and past testing experience for HIV, chlamydia, and gonorrhea.

	Subjects, n (%)	Probability difference^a^	One-sided 95% CI^b^	Intraclass correlation	Adjusted probability difference^a^	One-sided 95% CI^b^
Pay-it-forward group (n=240)	197 (82.1)	77.9%	70.3%	<0.001	76.7%	70.8%
Standard-of-care group (n=240)	10 (4.2)	N/A^c^	N/A	<0.001	N/A	N/A

^a^Indicates probability of difference between the intervention arm (pay-it-forward) and the standard-of-care arm.

^b^The lower-bound 95% CI is reported.

^c^N/A: not applicable.

**Table 3:** Subgroup analysis of 480 participants in China in 2020.

		SOC^a^ (n=240), n/N (%)	PIF^b^ (n=240), n/N (%)	Probability difference (PIF minus SOC), % (95% CI)	Difference in probability difference, % (95% CI)		*P* value	
**Age (years)**						11.2 (–0.6 to 23.1)^c^		.07
	≤30	3/81 (4)	64/86 (74)	70.7 (60.6-80.8)				
	>30	7/159 (4)	133/154 (86)	82 (75.7-88.2)				
**Condom use during commercial sex in the past 3 months**						–15 (–25.8 to –4.1)^d^		.007
	Inconsistent	6/167 (4)	100/134 (75)	71 (63.1-78.9)				
	Consistent	4/73 (5)	97/106 (92)	86 (78.6-93.5)				
**Tested for HIV in the past**						4 (–8.6 to 16.6)^e^		.54
	No	0/95 (0)	159/195 (82)	81.5 (76.1-87)				
	Yes	10/145 (7)	38/45 (84)	77.5 (66.2-88.9)				
**Workplace**						–5.1 (–16.3 to 6)^f^		.37
	High tier	5/157 (3)	122/154 (79)	76 (69.1-83)				
	Low tier	5/83 (6)	75/86 (87)	81 (72.5-89.9)				

^a^SOC: standard of care.

^b^PIF: pay it forward.

^c^≤30 years vs >30 years.

^d^Inconsistent vs consistent.

^e^No vs yes.

^f^High tier vs low tier.

### Economic Evaluation

Among 197 women who received chlamydia and gonorrhea tests in the pay-it-forward arm, 99 (50.3%) donated to the pooled funds. The total donation amount was US $326, and the median donation amount per donor was US $1.54 (IQR 0.77-1.54). The largest donation was US $119.54, and the lowest was US $0.15. Complete cost and cost-effectiveness analyses are provided in Multimedia Appendix 4, Tables S2 to S3 and Figures S1 to S3. The economic cost per person tested was US $42.24 for the standard-of-care arm and US $41.80 for the pay-it-forward arm. The ICER for the pay-it-forward arm compared to standard of care was minus US $2.79 per additional person tested.

## Discussion

### Principal Findings

Upstream reciprocity theory suggests that people who are cared for by someone are more likely to help others. Our study confirmed this theory by promoting chlamydia and gonorrhea testing among FSWs in China through a pay-it-forward approach. Our study extends the existing literature by testing the effectiveness of the pay-it-forward approach in increasing STI testing uptake among FSWs, conducting the study in an outreach setting outside of a formal clinic, and integrating the approach with HIV services. As a social innovation approach, we found that the pay-it-forward intervention dramatically increased STI testing uptake among Chinese FSWs and that the participants covered a substantial portion of the costs associated with testing.

Women in the pay-it-forward arm had higher chlamydia and gonorrhea testing uptake than women in the standard-of-care arm. This finding is consistent with previous studies among Chinese MSM, which suggest that the pay-it-forward approach can motivate more people to use STI testing services [10,17]. In addition, the pay-it-forward strategy is more effective than other STI testing–uptake intervention approaches, such as social marketing [23], digital health [24], and point of care [25]. Several factors may have led to the strong testing uptake among individuals in the pay-it-forward arm. The first was generosity and trust. Due to stigma and other barriers, few Chinese FSWs are willing to undergo or pay for chlamydia and gonorrhea testing [26]. The low testing rates in our standard-of-care arm demonstrate this. Our pay-it-forward strategy revealed substantial generosity and promoted responsibility among the FSWs to change their testing behaviors. Second, free testing may have driven the increased test uptake rates because of the zero-price effect [27]. Our previous studies indicate that generosity (ie, voluntary donations) integrated with free testing strongly motivates behavior change [10,17].

Lack of community engagement and low social trust are 2 critical barriers to STI testing uptake among FSWs [28]. In our study, about half of the FSWs who were tested in the pay-it-forward arm donated some money for future testing. Although the donation rate in our study was lower than in our previous studies among MSM in China [10,17], around half of the FSWs chose to contribute. This is remarkable given the low socioeconomic status of FSWs and the lack of a culture of donating in China. Pay-it-forward approaches may increase community ownership and social trust, supporting STI testing [16,29]. Two systematic reviews further support this explanation, reporting that being kind is associated with well-being, especially psychological functioning [30] and subjective well-being [31]. Qualitative studies that aim to further confirm these psychological impulses are needed.

Our findings indicate that the pay-it-forward strategy increased STI testing uptake and reduced barriers to sustaining STI testing. STI testing among FSWs reduces STI transmission and adverse sexual and reproductive health consequences. The pay-it-forward approach provides an alternative to using limited public funding to maintain or expand testing. Our findings need to be extended beyond this initial research study, as they have several public health implications. First, a pay-it-forward strategy could reduce costs for STI testing for funding bodies and dramatically remove the financial barrier to STI testing among participants. Second, unlike the free-testing approach, the pay-it-forward method could generate strong community ownership and trust. Third, as an approach to increase testing uptake and the regularity of testing, the pay-it-forward intervention has a robust public health benefit. Improving testing coverage and the regularity of testing could reduce the high chlamydia and gonorrhea prevalence among Chinese FSWs (17.3% for chlamydia in our study) by identifying and treating asymptomatic infections earlier, thus reducing morbidity and onward transmission to the clients of FSWs and their other sexual partners [32].

### Limitations and Implications

Our study has several limitations. First, although this was a cluster RCT, the number of clusters in our study was relatively small. Therefore, the characteristics of the participants in the 2 study arms were not completely balanced, which may have resulted in a selection bias. However, our main findings were robust when we adjusted for marital status, condom use during the last commercial sex act, and past HIV testing experience. In addition, the data were analyzed with GEE models for a small number of clusters. At the same time, a sensitivity analysis was also conducted by treating FSWs recruited from the same venue as a cluster. Second, the test uptake rate in the standard-of-care arm was low, further complicating data analysis. However, FSW test uptake rates are generally low in China [11] and many other LMICs [33]. Third, the level of community engagement in our study was suboptimal. Unlike our previous studies evaluating the pay-it-forward strategy in improving STI care, the FSWs in this study did not engage in community activities, such as passing postcards or messages to other FSWs in their social network; this needs to be improved in future studies.

Our study has several implications. First, qualitative research is needed to evaluate how and why generosity works among FSWs in promoting STI testing. Second, this study did not collect data on implementing the evidence-based pay-it-forward intervention and scaling it up. Additional implementation-science research is needed to expand the pay-it-forward intervention into other settings, including integrating it with other STI services. Third, strategies to increase demand and the informational messages on the need for chlamydia and gonorrhea screening (ie, the introduction to chlamydia and gonorrhea and the pay-it-forward intervention model) were not developed together with the FSWs; the study was carried out solely by study staff from public health authorities. Future research could examine how the engagement of local FSWs at the formative research stage could further improve testing uptake and program sustainability. Fourth, this study was implemented during the period from August to November 2020, a time when the COVID-19 pandemic may have impacted the implementation of the study itself and reduced the responsiveness of the FSWs, as many FSWs had temporarily ceased working during the pandemic period. We anticipated that more low-income FSWs would continue their work even while high-tier venues were shut down due to COVID-19 measures.

### Conclusion

Innovative and effective intervention strategies are urgently needed to fill the gap between the high global burden of chlamydia and gonorrhea disease and suboptimal STI testing services. A pay-it-forward approach has the potential to enhance chlamydia and gonorrhea testing uptake among Chinese FSWs. It may be a valuable tool for scaling up STI services. Further implementation research is needed to inform the transition of pay-it-forward research into practice.

## Appendix

Multimedia Appendix 1 CONSORT 2010 checklist.

Multimedia Appendix 2 Sensitivity analysis of number of cluster size.

Multimedia Appendix 3 Cost effectiveness analysis.

Multimedia Appendix 4 The reason for a test or not in the PIF program and the program's perceived benefits.

## Abbreviations

FSW: female sex worker
GEE: generalized estimating equations
ICER: incremental cost-effectiveness ratio
LMICs: low- and middle-income countries
MSM: men who have sex with men
RCT: randomized controlled trial
STI: sexually transmitted infection

## Data Availability

The deidentified data have been uploaded as supplemental data.
