# Supplementary material for: A Pay-It-Forward Approach to Improve Chlamydia and Gonorrhea Testing Uptake Among Female Sex Workers in China: Venue-Based Superiority Cluster Randomized Controlled Trial
Source: JMIR Public Health Surveill. 2023 Mar 2;9:e43772. doi: 10.2196/43772 (PMC10020898; doi:10.2196/43772)
Supplement: Multimedia Appendix 2 [file publichealth_v9i1e43772_app2.docx]

## **Multimedia Appendix 1: Sensitivity analysis of number of cluster size**

Table S1. The proportion of participating FSWs tested for chlamydia and gonorrhea in Guangdong, China, in 2020 (N=480, cluster defined as FSWs workplace).

|  | **n/N (%)** | **Number of**  **groups** | **Probability difference* (%)** | **One-side 95% CI^ǂ^** | **Intraclass**  **correlation** | **Adjusted probability difference*(%)** | **One-side 95% CI^ǂ^** |
| --- | --- | --- | --- | --- | --- | --- | --- |
| **Pay-it-forward group** | 197/240 (82·1) | 8 | 77·9 | 68.9 | 0.002 | 76.9 | 63.7 |
| **Standard of care group** | 10/240 (4·2) | 8 | - | - | 0.126 | - | - |
| ***The probability of difference between the intervention arms (Pay-it-forward) and standard of care.**  **^ǂ^A lower bound 95% confidence interval was reported.**  **^#^ Due to sampling limitation, model only adjusted for marital status to achieve convergence.** | | | | | | | |
