# Supplementary material for: A Pay-It-Forward Approach to Improve Chlamydia and Gonorrhea Testing Uptake Among Female Sex Workers in China: Venue-Based Superiority Cluster Randomized Controlled Trial
Source: JMIR Public Health Surveill. 2023 Mar 2;9:e43772. doi: 10.2196/43772 (PMC10020898; doi:10.2196/43772)
Supplement: Multimedia Appendix 3 [file publichealth_v9i1e43772_app3.docx]

## **Multimedia Appendix 3: The reason for a test or not in the PIF program and the program's perceived benefits.**

Most women who participated in the pay-it-forward arm and received testing reported that their primary reason for testing was because the researcher introduced chlamydia and gonorrhea testing. The most common reasons for not testing in the pay-it-forward arm were "I don't know about chlamydia and gonorrhea.". The top three benefits of the intervention arm were: "other participants paid the testing fee and reduced the financial burden," "here provided chlamydia and gonorrhea testing services," and "more female sex workers can be tested for chlamydia and gonorrhea through the strategy" (Table S4).

| **Table S4. The reason for a test or not in the PIF program and the program's perceived benefits.** | |
| --- | --- |
| **The main reasons for chlamydia and gonorrhea testing in the PIF program** | **FSW who choose the following answers** |
| Because the researcher introduced chlamydia and gonorrhea testing | 117/197 (59·4) |
| I have some clinical symptoms recently | 1/197 (0·5) |
| I had high-risk sex recently | 20/197 (10·2) |
| Friends/family asked me to test | 5/197 (2·5) |
| The medical staff asked me to test | 40/197 (20·3) |
| The PIF program | 13/197 (6·6) |
| **Reason for not participating in the PIF program (Multiple options)** | **FSW who choose the following answers** |
| I don't know about chlamydia and gonorrhea | 24/43 (55·8) |
| I don't want to know if I have gonorrhea or chlamydia | 9/43 (20·9) |
| I don't need to be tested | 2/43 (4·7) |
| Too much trouble | 4/43 (9·3) |
| I am worried that my privacy will be leaked | 15/43 (34·9) |
| I am afraid of pain | 4/43 (9·3) |
| I am ashamed to let medical staff take samples | 3/43 (7·0) |
| I am embarrassed to have a test in front of a friend or partner | 4/43 (9·3) |
| I am afraid that the test result is already infected | 9/43 (20·9) |
| I had tested recently | 4/43 (9·3) |
| **Benefits of participating in the PIF program (Multiple options)** | **FSW who choose the following answers** |
| Other participants paid the testing fee and reduced my financial burden | 154/197 (78·2) |
| Let us know that there are chlamydia and gonorrhea testing services | 108/197 (54·8) |
| More sex workers can be tested for chlamydia and gonorrhea | 93/197 (47·2) |
| Can reduce the spread of chlamydia and gonorrhea in the population | 77/197 (39·1) |
| Feel and convey everyone's care and warmth | 80/197 (40·6) |
