# Supplementary material for: A Pay-It-Forward Approach to Improve Chlamydia and Gonorrhea Testing Uptake Among Female Sex Workers in China: Venue-Based Superiority Cluster Randomized Controlled Trial
Source: JMIR Public Health Surveill. 2023 Mar 2;9:e43772. doi: 10.2196/43772 (PMC10020898; doi:10.2196/43772)

## **Multimedia Appendix 2: Cost effectiveness analysis**

**Costing analysis**

We used a microcosting method to assess the whole economic cost (i. e., the cost of all resources needed to implement the testing models) from the perspective of a health provider. We kept track of the resources utilized throughout the experiment and classified cost items as fixed or variable. We assessed the start-up (training) and personnel compensation expenses for the outreach team as fixed costs (i.e. regardless on the number of tests completed). We evaluated the cost of supplies used for chlamydia and gonorrhea testing as variable costs (i.e. based on the number of tests completed). All expenses are expressed in 2020 USD using OANDA currency conversions (1USD = 6.50 Yuan). We conducted a cost analysis in Excel 2019 (Microsoft, USA).

**Cost-effectiveness analysis**

Using parameters from Table S2, which were informed by the trial, we created a decision-tree model using TreeAge Pro 2020 (TreeAge Software Inc) to explore the cost-effectiveness of pay-it-forward and the standard-of-care. The decision tree model is in Figure S1.

Among 197 women who received chlamydia and gonorrhea tests in the pay-it-forward arm, 99 (50·3%) donated to the pooled funds. The total donation amount was $326, and the median donation amount per donor was $1·54 (IQR:0·77-1·54). The largest donation was $119·54, and the lowest was $0·15.

The incremental cost for each treatment group and the incremental cost-effectiveness ratios (ICERs) based on financial and economic costs, respectively, are shown in Table S3. The PIF strategy was found cheaper and more effective than the SOC in promoting testing and case identification among FSWs. Results from the univariate sensitivity analyses are presented as tornado plots in Figure S2. For ICERs of Economic/financial cost per additional person tested, the biggest drivers of cost-effectiveness were the variable costs of supplies of PIF group when comparing PIF with SOC (Figure S2). Probabilistic sensitivity analyses with 100,000 runs were conducted and presented as cost-effectiveness acceptability curves. Figure S3.1 shows that the PIF has a greater probability of being more cost-effectiveness than SOC if the willingness to pay is greater than $20 per person tested.

| **Table S2. Unit costs of pay-it-forward and standard of care in the model.** | | |
| --- | --- | --- |
| **Tested performed** | **Probability per person tested** | **Distribution** |
| PIF* | 0.82 | Beta (47.47, 134.80) |
| SOC* | 0.04 | Beta (16.60, 381.71) |
| **Chlamydia or gonorrhea diagnosed** |  |  |
| PIF | 0.18 | Beta (30.14, 134.80) |
| SOC | 0 | Beta (0, 0) |
| **Fixed cost PIF or SOC** | **USD 2020** |  |
| Start-up | 0.95 | Gamma (40.19, 42.27) |
| Personnel | 0.26 | Gamma (41.82, 161.67) |
| **Variable cost PIF** |  |  |
| Supplies | 14.07 | Gamma (42.84, 3.04) |
| **Variable cost SOC** |  |  |
| Supplies | 14.52 | Gamma (42.76, 2.95) |
| **Donations/payment** |  |  |
| PIF | 2.69 | Gamma (43.11, 16.01) |
| SOC | 0.96 | Gamma (41.14, 42.76) |
| *PIF=pay-it-forward, SOC= standard of care | | |

**Table S3. Outcome analyses for costs of each group**

| Treatment group | Economic cost (USD) | Incremental cost | Probability of people tested | Positive cases (NG/ CT) | ICER (USD per person CT/NGtested) | ICER (USD per CT/NGcase identified) |
| --- | --- | --- | --- | --- | --- | --- |
| SOC | 42.24 | - | 0.04 | 0 | - | - |
| PIF | 41.80 | -0.44 | 0.82 | 0.18 | Dominated* | Dominated* |
| Treatment group | Financial cost (USD) | Incremental cost | Number of people tested | Positive cases (NG/ CT) | ICER (USD per person CT/NG tested) | ICER (USD per CT/NG case identified) |
| SOC | 41.28 | - | 0.04 | 0 | - | - |
| PIF | 39.10 | -2.17 | 0.82 | 0.18 | Dominated* | Dominated* |

ICER = incremental cost-effectiveness ratio; PIF = pay-it-forward; SOC = standard of care; USD = United States dollars

* A dominated strategy is cheaper and more effective than the comparator (SOC).

**Figure S1. Decision tree model.**

**
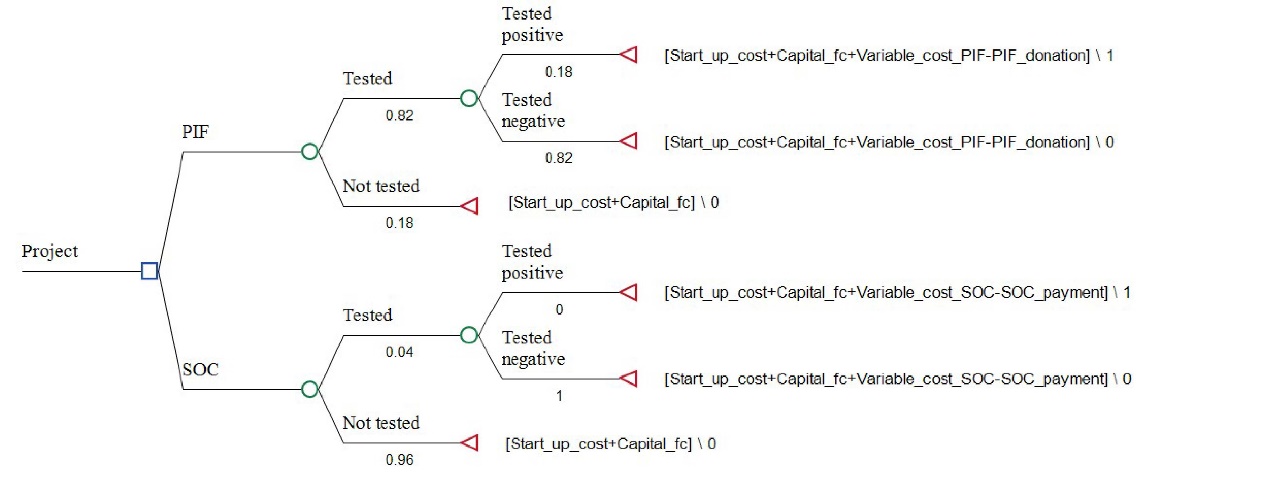
**

*Start_up_cost:The training cost to start the study; Capital_fc: The capital fixed cost of the study; Variable_cost_PIF/SOC: variable cost in the PIF/SOC group, variable cost means the fees of sample collection, transportation and testing; PIF_donation: The money donated to the PIF program by the participants; SOC_payment: The fees paid for the testing service by participants in the SOC; SOC_testing: The probability of participants tested in the SOC; PIF_testing: The probability of participants tested in the PIF; PIF_positive: The probability of participants tested positive in the PIF.*

**Figure S2. Univariate analysis for comparing the cost-effectiveness of PIF vs. SOC (financial cost).**


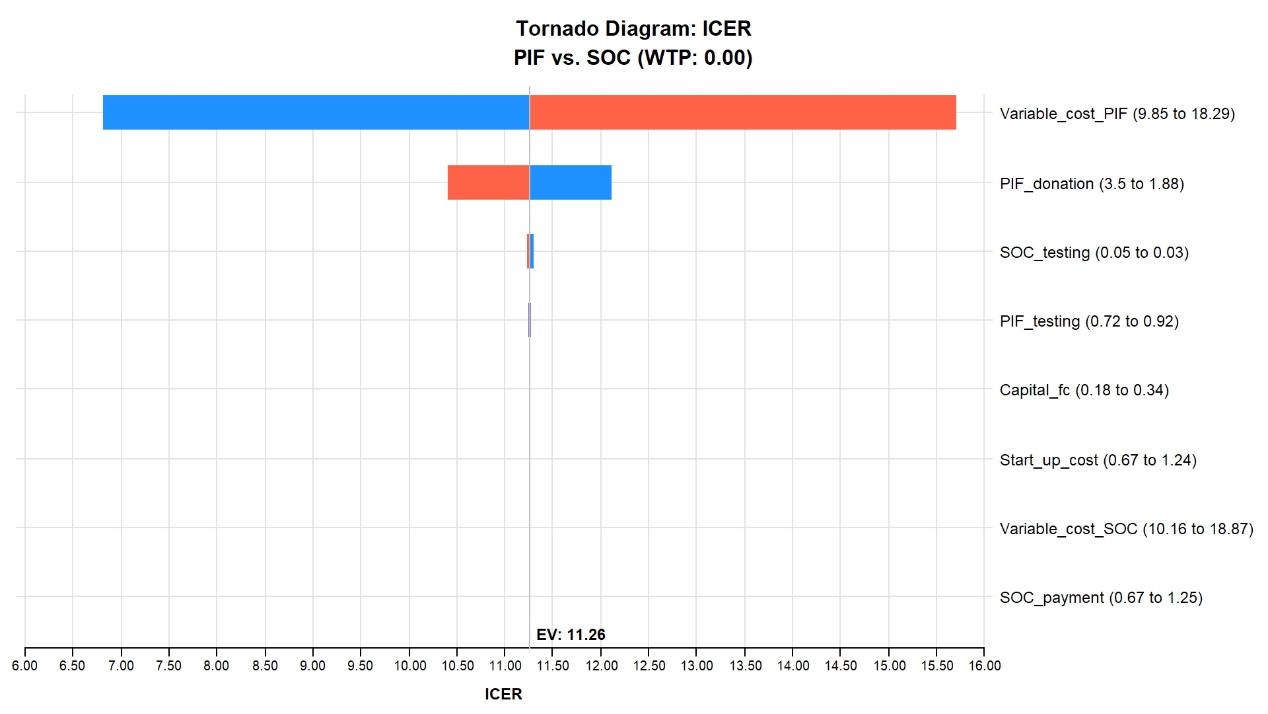


*Start_up_cost:The training cost to start the study; Capital_fc: The capital fixed cost of the study; Variable_cost_PIF/SOC: variable cost in the PIF/SOC group, variable cost means the fees of sample collection, transportation and testing; PIF_donation: The money donated to the PIF program by the participants; SOC_payment: The fees paid for the testing service by participants in the SOC; SOC_testing: The probability of participants tested in the SOC; PIF_testing: The probability of participants tested in the PIF; PIF_positive: The probability of participants tested positive in the PIF.*

**Figure S3.1. Cost-effectiveness acceptability curve of the financial cost per person tested.**
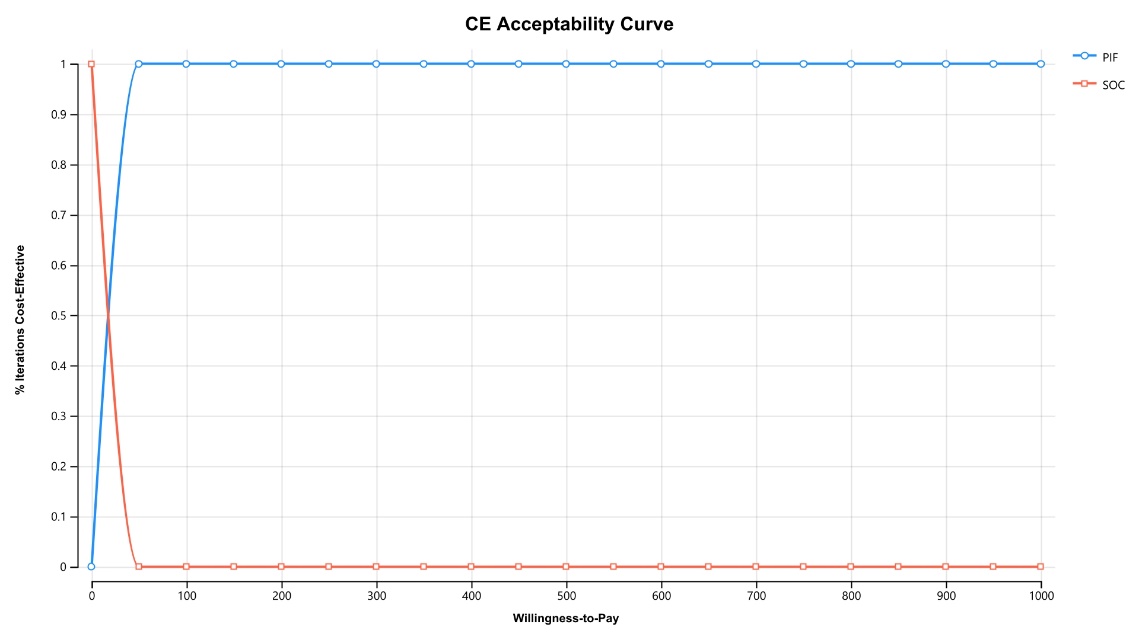

Supplement: Multimedia Appendix 4 [file publichealth_v9i1e43772_app4.docx]
